# National, regional, and state-level pneumonia and severe pneumonia morbidity in children in India: modelled estimates for 2000 and 2015

**DOI:** 10.1016/S2352-4642(20)30129-2

**Published:** 2020-09

**Authors:** Brian Wahl, Maria Deloria Knoll, Anita Shet, Madhu Gupta, Rajesh Kumar, Li Liu, Yue Chu, Molly Sauer, Katherine L O'Brien, Mathuram Santosham, Robert E Black, Harry Campbell, Harish Nair, David A McAllister

**Affiliations:** a International Vaccine Access Center, Baltimore, MD, USA; b Institute for International Programs, Baltimore, MD, USA; c Department of Population, Family and Reproductive Health, Johns Hopkins Bloomberg School of Public Health, Baltimore, MD, USA; d Department of Community Medicine and School of Public Health, Postgraduate Institute of Medical Education and Research, Chandigarh, India; e Department of Sociology, Institute for Population Research, Ohio State University, Columbus, OH, USA; f Centre for Global Health, Usher Institute, University of Edinburgh, Edinburgh, UK; g Public Health Foundation of India, New Delhi, India; h Institute of Health and Wellbeing, University of Glasgow, Glasgow, UK; i World Health Organization, Geneva, Switzerland

## Abstract

**Background:**

The absolute number of pneumonia deaths in India has declined substantially since 2000. However, pneumonia remains a major cause of morbidity in children in the country. We used a risk factor-based model to estimate pneumonia and severe pneumonia morbidity in Indian states in 2000 and 2015.

**Methods:**

In this modelling study, we estimated the burden of pneumonia and severe pneumonia in children younger than 5 years using a risk factor-based model. We did a systematic literature review to identify published data on the incidence of pneumonia from community-based longitudinal studies and calculated summary estimates. We estimated state-specific incidence rates for WHO-defined clinical pneumonia between 2000 and 2015 using Poisson regression and the prevalence of risk factors in each state was obtained from National Family Health Surveys. From clinical pneumonia studies, we identified studies reporting the proportion of clinical pneumonia cases with lower chest wall indrawing to estimate WHO-defined severe pneumonia cases. We used the estimate of the proportion of cases with lower chest wall indrawing to estimate WHO-defined severe pneumonia cases for each state.

**Findings:**

Between 2000 and 2015, the estimated number of pneumonia cases in Indian HIV-uninfected children younger than 5 years decreased from 83·8 million cases (95% uncertainty interval [UI] 14·0–300·8) to 49·8 million cases (9·1–174·2), representing a 41% reduction in pneumonia cases. The incidence of pneumonia in children younger than 5 years in India was 657 cases per 1000 children (95% UI 110–2357) in 2000 and 403 cases per 1000 children (74–1408) in 2015. The estimated national pneumonia case fatality rate in 2015 was 0·38% (95% UI 0·11–2·10). In 2015, the estimated number of severe pneumonia cases was 8·4 million (95% UI 1·2–31·7), with an incidence of 68 cases per 1000 children (9–257) and a case fatality ratio of 2·26% (0·60–16·30). In 2015, the estimated number of pneumonia cases in HIV-uninfected children was highest in Uttar Pradesh (12·4 million [95% UI 2·1–45·0]), Bihar (7·3 million [1·3–26·1]), and Madhya Pradesh (4·6 million [0·7–17·0]). Between 2000 and 2015, the greatest reduction in pneumonia cases was observed in Kerala (82% reduction). In 2015, pneumonia incidence was greater than 500 cases per 1000 children in two states: Uttar Pradesh (565 cases per 1000 children [95% UI 94–2047]) and Madhya Pradesh (563 cases per 1000 children [88–2084]).

**Interpretation:**

The estimated number of pneumonia and severe pneumonia cases among children younger than 5 years in India decreased between 2000 and 2015. Improvements in socioeconomic indicators and specific government initiatives are likely to have contributed to declines in the prevalence of pneumonia risk factors in many states. However, pneumonia incidence in many states remains high. The introduction of new vaccines that target pneumonia pathogens and reduce risk factors will help further reduce the burden of pneumonia in the country.

**Funding:**

Bill & Melinda Gates Foundation.

## Introduction

Efforts to improve access to primary health services in India have contributed to substantial reductions in pneumonia mortality in the past decade; however, pneumonia remains the leading cause of mortality in children after the neonatal period in the country.^1^ In 2015, there were an estimated 192 000 pneumonia deaths in children younger than 5 years, representing a 57% reduction in pneumonia mortality since 2000.^1^ As deaths attributed to pneumonia continue to decline, reducing the burden of pneumonia morbidity through primary interventions in particular will become increasingly important. Updated subnational estimates of pneumonia morbidity can help to monitor progress in this regard.

WHO has identified several interventions for the prevention of pneumonia in children.^2^ Many of these interventions are associated with improving nutrition and reducing exposure to environmental and social risk factors, including exclusive breastfeeding for the first 6 months of life, adequate complementary feeding, vitamin A supplementation, handwashing with soap, reducing exposure to indoor air pollution, and immunisation.^2^ The urgent need to focus on pneumonia prevention is compounded by the increasing prevalence of antibiotic resistance that threatens to make the treatment of bacterial causes of pneumonia more challenging and expensive in countries such as India.^3^

**Research in context**

**Evidence before this study**

We searched PubMed for modelled national and subnational estimates of pneumonia and severe pneumonia morbidity in children in India published between Jan 1, 2000, and Dec 31, 2018, using the search terms “pneumonia”, “respiratory infection”, “India”, and “model”. We included estimates of lower respiratory infections and severe lower respiratory infections in our search. Several national estimates of pneumonia and severe pneumonia morbidity have been published by the Child Health Epidemiology Reference Group collaboration (now the WHO Maternal and Child Epidemiology Estimation collaboration). Based on the most contemporary of these estimates published in 2018, there were approximately 45 million cases of pneumonia and 7 million cases of severe pneumonia in 2015. National and subnational estimates of severe pneumonia morbidity for 2010 were published in 2015. Based on these estimates, there were 3·6 million severe pneumonia cases in India in 2010. In the same paper, the researchers published estimates of deaths due to pneumonia in 2010, corresponding to a case fatality ratio of approximately 1% for children younger than 5 years.

**Added value of this study**

To our knowledge, our findings represent the most up to date estimates of national pneumonia and severe pneumonia morbidity in India. Although estimates of severe pneumonia cases in India have been published previously, we believe that these are also the first subnational estimates for pneumonia and severe pneumonia to be presented together. Additionally, we have updated our risk factor prevalence-based model used to derive pneumonia case estimates. Our updated model uses seven risk factors for pneumonia instead of the five or six risk factors used in previous models. Earlier models assumed that pneumonia risk factors were independent. The completeness of the India survey data allowed us to directly measure the number of children with each combination of risk factors using individual-level survey data, which enabled us to build the risk factor model using the observed correlation between risk factors.

**Implications of all the available evidence**

These updated estimates reflect the reductions in pneumonia and severe pneumonia morbidity in India since 2000, as a result of changes in the prevalence of seven pneumonia risk factors. These data, together with all the available evidence, should be used to inform policies and programmes that target pneumonia morbidity in India. Additionally, the methods developed for this study can be used as a framework to model data from other settings.

Aetiological contributors to pneumonia morbidity globally include *Streptococcus pneumoniae* (pneumococcus), *Haemophilus influenzae* type b (Hib), respiratory syncytial virus, influenza, and human metapneumovirus.^4^, ^5^ India introduced the Hib-containing pentavalent vaccine in two states in 2011.^6^ According to the International Vaccine Center's VIEW-hub, the vaccine is now used routinely in all states and union territories in India. The 13-valent pneumococcal conjugate vaccine was introduced in three states in 2017, which was expanded to three additional states in 2018 with plans to expand its routine use in coming years.^5^ The Indian Government is also currently implementing other programmes that could reduce the burden of pneumonia in India, including the Integrated Child Development Services scheme that aims to improve the nutritional status of children^7^ and the Mothers' Absolute Affection programme, which promotes exclusive breastfeeding during the first 6 months of life.^8^

Previous national and subnational estimates of pneumonia morbidity for India have been published.^9^, ^10^ Using subnational input data, researchers estimated there were 3·6 million severe pneumonia cases in 2010 in children younger than 5 years in India.^10^ This model used a risk factor-based approach originally developed by WHO and the Maternal and Child Epidemiology Estimation (WHO/MCEE) collaboration.^11^ Using a similar approach, the WHO/MCEE estimated that nationally there were 45·0 million pneumonia cases and 7·2 million severe pneumonia cases among children in India in 2015.^12^ For simplicity, these models assumed that the prevalence of pneumonia risk factors were independent—eg, children who had a low birthweight are no more likely to have malnutrition than children who did not have low birthweight. To support policy making associated with child health interventions in India, we used a method that instead uses the observed risk factor combinations to calculate pneumonia and severe pneumonia morbidity in Indian states between 2000 and 2015.

## Methods

### Literature review

We estimated the burden of pneumonia and severe pneumonia using a risk factor-based model, which has been described previously.^12^ Briefly, we used data from a 2018 systematic literature review that identified published data on the incidence of pneumonia from community-based longitudinal studies around the world to identify summary estimates of risk factors associated with pneumonia in children.^12^ Only studies that reported the incidence of WHO-defined clinical cases of pneumonia in children younger than 5 years and those that were done for a minimum of 1 year and in multiples of 12 months to account for seasonal variations in incidence were included in the model. From clinical pneumonia studies, the earlier review identified studies that reported the proportion of clinical pneumonia cases with lower chest wall indrawing to estimate WHO-defined severe pneumonia cases. For pneumonia risk factors, we used summary estimates from a previous review^13^ that identified factors associated with pneumonia (table 1). Details of the literature review are listed in the appendix (pp 3–5).

**Table 1:** Model parameters, data sources, and summary estimates

	**Data sources**	**Summary estimate**
**Pneumonia and severe pneumonia morbidity**		
Baseline pneumonia incidence in the community (per child-year)	Published summary estimate^12^	0·22 (IQR 0·11–0·51)
Proportion of severe pneumonia cases	Published summary estimate^12^	11·5% (IQR 8·0–33·0)
**Pneumonia risk factors**		
Low birthweight (<2·5 kg)	Published summary estimate^13^	OR 3·6 (95% CI 0·8–16·3)
Lack of exclusive breastfeeding for the first 6 months of life	Published summary estimate^13^	OR 2·7 (95% CI 1·7–4·4)
Crowding (seven or more individuals per household)	Published summary estimate^13^	OR 1·9 (95% CI 1·5–2·5)
Indoor air pollution exposure	Published summary estimate^13^	OR 1·6 (95% CI 1·1–2·3)
Malnutrition (weight-for-age ≤2 SDs)	Published summary estimate^13^	OR 4·5 (95% CI 2·1–9·5)
Incomplete immunisation at 12 months	Published summary estimate^13^	OR 1·8 (95% CI 1·3–2·5)
HIV infection	Published summary estimate^14^	OR 6·5 (95% CI 5·9–7·2)
**Population at risk and demographic model parameters**		
Child population	2001 and 2011 census data from the Government of India	State-specific values
Hib vaccine coverage	Inferred from DTP3 coverage estimates from AHS, DLHS, and NFHS	State-specific values
Pneumonia mortality	Estimates of pneumonia mortality from WHO/MCEE collaboration^1^	State-specific values

OR=odds ratio. Hib=*Haemophilus influenzae* type b. DTP3=third dose of diphtheria–tetanus–pertussis vaccine. AHS=Annual Health Survey. DLHS=District Level Household and Facility Survey. NFHS=National Family Health Survey. WHO/MCEE=WHO and the Maternal Child Epidemiology Estimation.

### Data analysis

Model parameters and data sources are shown in table 1. We obtained data on the prevalence of identified risk factors (with the exception of HIV infection) in each state from the National Family Health Survey, which uses standardised Demographic and Health Survey data collection methods.^15^ This survey is intended to be done every 5 years in India and provides data on a range of demographic and health indicators. For small states that were not included in National Family Health Surveys, we used prevalence estimates from neighbouring states with similar demographics. The prevalence of HIV infection in children was estimated using the proportion of children in each state with mothers infected with HIV and the UNAIDS estimate of the odds ratio for HIV infection in children born to mothers with HIV infection in India. We used India census data from 2001 and 2011 to estimate annual population growth rates for each state, which were extrapolated to 2015.^16^ We normalised population data to sum to the total national UN population estimates for India.

We estimated state-specific pneumonia incidence by combining overall incidence estimates from published papers for the WHO South-East Asia region, published effect estimates for associations between risk factors and pneumonia, and risk factor prevalence data from state-level survey data. We combined each of these estimates in a risk-factor based model, sampling from our previous model,^12^ log-normal distributions, and Dirichlet distributions for the regional incidence, risk factor associations, and survey data. We also calculated the incidence of clinical pneumonia in children with HIV and incidence of pneumonia attributable to HIV. We applied the estimate of the proportion of cases with lower chest wall indrawing to state-level WHO-defined clinical pneumonia cases for each state. Additional details about the model are in the appendix (pp 3–5).

After calculating pneumonia and severe pneumonia cases for each state, we used estimates of Hib vaccine efficacy against invasive Hib disease,^17^ the proportion of clinical pneumonia cases attributable to Hib,^18^ and state-level vaccine coverage estimates in a post-hoc adjustment to account for the proportion of children who would have been protected by the Hib vaccine. We used coverage of the third dose of diphtheria–tetanus–pertussis vaccine from subnational surveys for coverage with three doses of Hib vaccine. This approach is justified because Hib vaccine, when and where used, is provided as part of a pentavalent combination that includes diphtheria–tetanus–pertussis vaccination in the national immunisation programme. We adjusted coverage estimates to account for the time of year that Hib vaccine was introduced. Linear interpolation was used when data were missing for relevant years. Pneumococcal conjugate vaccine was only available in India in the private sector in 2015 with low estimated population-level coverage.^19^ Therefore, we assumed pneumococcal conjugate vaccine had no impact on pneumonia morbidity in children before 2015. We used published state-level estimates of pneumonia deaths in children from 2015^1^ to independently infer case fatality ratios.

Using state-level estimates of pneumonia mortality for 2015 published by the WHO/MCEE collaboration,^1^ we estimated pneumonia and severe pneumonia case fatality ratios in all states in 2000 and 2015.

We used a simulation approach to calculate state-specific rates and corresponding uncertainty estimates, presented as uncertainty intervals (UIs; appendix p 5). All statistical analyses were done using R (version 3.2) and JAGS (version 3.4) software. Code for the updated model is available online.

### Reporting

For the purpose of this analysis, we report pneumonia and severe pneumonia morbidity estimates for six geographically contiguous and socioeconomically similar regions: north, east, northeast, central, west, and south. For state-level reporting, union territories, with the exception of Delhi, and states in the northeast, with the exception of Assam, have been grouped together. Telangana separated from Andhra Pradesh in June, 2014. We projected population and mortality data for Andhra Pradesh and modelled the pneumonia and severe pneumonia burden for Andhra Pradesh and Telangana together for 2015. We report on the pneumonia and severe pneumonia morbidity together for nine states with high infant mortality that together have been designated as high-focus states by the Government of India (table 2). The state and regional estimates are related to the total estimate only through the estimated rate in the unexposed group (ie, children without pneumonia risk factors). The state counts will therefore not sum to the national counts. This study was done in accordance with the Guidelines for Accurate and Transparent Health Estimates Reporting (GATHER) recommendations (appendix p 2).^20^

**Table 2:** Indian states by region and categories

	**States**
Central	Chhattisgarh, Madhya Pradesh, Rajasthan, and Uttar Pradesh
East	Bihar, Jharkhand, Odisha, and West Bengal
North	Chandigarh, Delhi, Haryana, Himachal Pradesh, Jammu and Kashmir, Punjab, and Uttarakhand
Northeast*	Arunachal Pradesh, Assam, Manipur, Meghalaya, Mizoram, Nagaland, Sikkim, and Tripura
South	Andaman and Nicobar Islands, Andhra Pradesh, Karnataka, Kerala, Lakshadweep, Puducherry, and Tamil Nadu
West	Dadra and Nagar Haveli, Daman and Diu, Goa, Gujarat, and Maharashtra
Union territories	Andaman and Nicobar Islands, Chandigarh, Dadra and Nagar Haveli, Daman and Diu, Delhi, Lakshadweep, and Puducherry
High-focus states†	Assam, Bihar, Chhattisgarh, Jharkhand, Madhya Pradesh, Odisha, Rajasthan, Uttar Pradesh, and Uttarakhand

* Northeast states exclude Assam for state-level estimates.

† States with high infant mortality are designated as high-focus states by the Government of India.

### Role of the funding source

The funder of this study had no role in the study design, data collection, data analysis, data interpretation, writing of the report, or the decision to submit this report for publication. All authors had full access to all the data used in the study and the corresponding author had final responsibility for the decision to submit for publication.

## Results

In 2015, 49·8 million pneumonia cases (95% UI 9·1–174·2) were estimated to have occurred in HIV-uninfected children younger than 5 years in India. At the national level, pneumonia cases declined by 41% since 2000, when there were an estimated 83·8 million pneumonia cases (14·0–300·8) in this age group. Between 2000 and 2015, at the national level, pneumonia incidence in HIV-uninfected children younger than 5 years in India decreased by 39% from 657 cases per 1000 children (95% UI 110–2357) to 403 cases per 1000 children (74–1408). In 2015, there were an estimated 8·4 million (95% UI 1·2–31·7) cases of severe pneumonia in children younger than 5 years with a corresponding incidence rate of 68 cases per 1000 children (9–257).

In 2015, the pneumonia incidence in the central region in children younger than 5 years (540 cases per 1000 children [95% UI 89–1967]) was more than twice that of the incidence in the south region (251 cases per 1000 children [55–810]). The estimated decline in pneumonia incidence between 2000 and 2015 was highest in the north region (53%): pneumonia incidence in the west region reduced by 22% during the same time period. The central region also had the greatest estimated severe pneumonia incidence in children younger than 5 years (91 cases per 1000 children [95% UI 11–355]) in 2015.

In 2015, the estimated incidence of pneumonia in children younger than 5 years was higher in the high-focus states (513 cases per 1000 children [95% UI 87–1847]) than the non-high-focus states (331 cases per 1000 children [64–1134]). Pneumonia incidence in high-focus states declined by approximately the same amount as non-high-focus states between 2000 and 2015. In 2015, the incidence of severe pneumonia in high-focus states was 87 cases per 1000 children (95% UI 11–336) compared with 56 cases per 1000 children (8–208) in non-high-focus states.

**Figure 1:**
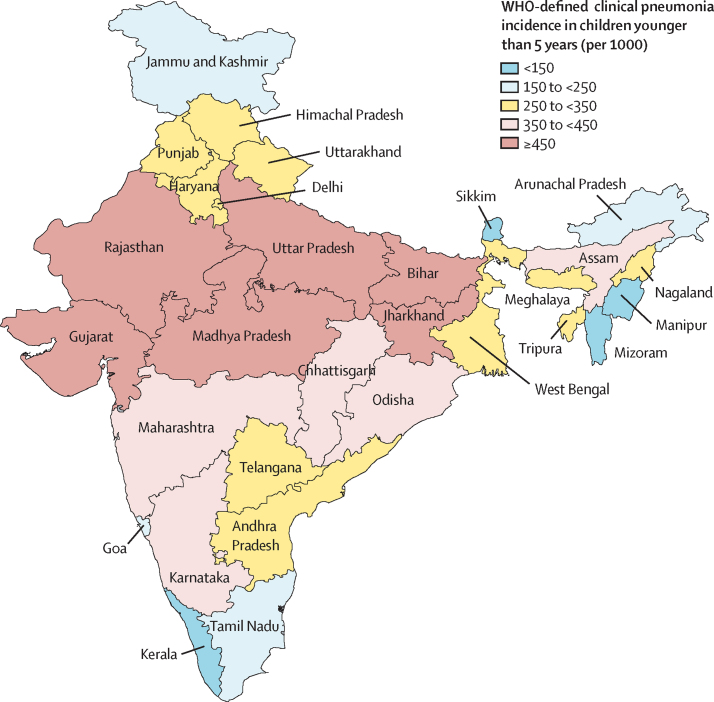
Incidence of WHO-defined clinical pneumonia in India in 2015 by state* *Andhra Pradesh and Telangana were calculated together.

The incidence and number of cases of pneumonia and severe pneumonia for each state in 2000 and 2015 are shown in table 3. In 2015, the highest estimated number of pneumonia cases in children uninfected with HIV occurred in Uttar Pradesh (12·4 million [95% UI 2·1–45·0]), Bihar (7·3 million [1·3–26·1]), and Madhya Pradesh (4·6 million [0·7–17·0]). In all states, the estimated number of pneumonia cases decreased between 2000 and 2015. The greatest reduction was observed in Kerala (73% reduction) and the smallest reduction was observed in Gujarat (18% reduction) during this time period. In 2015, pneumonia incidence was greater than 500 cases per 1000 children in two states: Uttar Pradesh (565 cases per 1000 children [95% UI 94–2047]) and Madhya Pradesh (563 cases per 1000 children [88–2084]; figure 1).

**Table 3:** National-level and state-level estimates of pneumonia and severe pneumonia morbidity in Indian children younger than 5 years

	**2000**				**2015**			
	Pneumonia cases* (95% UI)	Pneumonia incidence (95% UI)	Severe pneumonia cases* (95% UI)	Severe pneumonia incidence (95% UI)	Pneumonia cases* (95% UI)	Pneumonia incidence (95% UI)	Severe pneumonia cases* (95% UI)	Severe pneumonia incidence (95% UI)
**States**								
Andhra Pradesh and Telangana	3920 (700–13 680)	504 (90–1 758)	660 (90–2 510)	85 (11–323)	1830 (380–6110)	275 (57–918)	310 (50–1120)	46 (7–169)
Assam	2000 (340–7220)	574 (98–2068)	340 (40–1300)	97 (13–371)	1250 (250–4 230)	350 (69–1184)	210 (30–780)	59 (9–218)
Bihar	10 860 (1730–39 510)	865 (138–3148)	1840 (220–7250)	147 (18–578)	7330 (1290–26 060)	498 (87–1773)	1240 (160–4720)	84 (11–321)
Chhattisgarh	2520 (380–9320)	892 (136–3294)	430 (50–1710)	151 (17–603)	1120 (200–3960)	401 (71–1416)	190 (30–730)	68 (9–260)
Delhi	900 (150–3180)	566 (97–1997)	150 (20–580)	96 (13–364)	430 (80–1480)	285 (54–983)	70 (10–280)	48 (7–184)
Goa	70 (10–230)	542 (91–1943)	10 (0–40)	92 (12–356)	20 (0–70)	203 (44–667)	0 (0–10)	34 (6–124)
Gujarat	3620 (610–12 900)	596 (101–2123)	610 (80–2380)	101 (13–391)	2960 (500–10 770)	493 (83–1792)	500 (60–1960)	83 (11–326)
Haryana	1420 (240–5050)	540 (92–1926)	240 (30–930)	91 (12–353)	850 (170–2870)	326 (65–1100)	140 (20–530)	55 (8–205)
Himachal Pradesh	330 (60–1170)	502 (87–1801)	60 (10–210)	85 (11–327)	190 (40–650)	320 (60–1112)	30 (0–120)	54 (8–206)
Jammu and Kashmir	690 (110–2450)	642 (107–2282)	120 (10–450)	109 (14–422)	390 (90–1240)	218 (49–695)	70 (10–230)	37 (6–131)
Jharkhand	3240 (520–11 780)	865 (138–3148)	550 (70–2160)	147 (18–578)	1920 (340–6720)	464 (83–1625)	320 (40–1220)	78 (11–296)
Karnataka	3130 (560–10 690)	548 (99–1873)	530 (70–1990)	93 (12–349)	2000 (380–6820)	362 (69–1235)	340 (50–1260)	61 (9–228)
Kerala	1300 (250–4360)	402 (79–1346)	220 (30–800)	68 (10–248)	350 (100–930)	137 (40–365)	60 (10–180)	23 (5–69)
Madhya Pradesh	7600 (1150–28 050)	892 (136–3294)	1290 (150–5140)	151 (17–603)	4590 (720–16 990)	563 (88–2084)	780 (90–3060)	95 (12–375)
Maharashtra	6320 (1100–22 240)	572 (99–2012)	1070 (140–4060)	97 (13–368)	4350 (770–15 520)	431 (76–1538)	740 (100–2810)	73 (10–279)
Northeast	800 (140–2830)	540 (94–1900)	140 (20–520)	91 (12–351)	420 (100–1280)	248 (60–750)	70 (10–240)	42 (7–139)
Odisha	2570 (430–9140)	622 (104–2215)	430 (60–1680)	105 (13–408)	1580 (280–5540)	394 (71–1386)	270 (40–1020)	67 (9–254)
Punjab	1420 (230–5140)	576 (93–2086)	240 (30–950)	98 (12–385)	600 (120–2040)	258 (50–879)	100 (10–380)	44 (6–164)
Rajasthan	6930 (1090–25 310)	828 (130–3026)	1170 (140–4590)	140 (17–549)	3970 (650–14 520)	499 (81–1826)	670 (80–2630)	84 (10–331)
Tamil Nadu	2420 (460–8200)	411 (78–1395)	410 (60–1520)	70 (10–259)	980 (260–2850)	169 (44–491)	170 (30–540)	29 (6–92)
Union territories	150 (30–530)	526 (91–1859)	30 (0–100)	89 (12–342)	80 (20–260)	257 (54–857)	10 (0–50)	43 (7–158)
Uttar Pradesh	20 120 (3200–73 050)	838 (133–3041)	3410 (410–13 410)	142 (17–558)	12 410 (2070–44 960)	565 (94–2047)	2100 (270–8130)	96 (12–370)
Uttarakhand	890 (140–3240)	838 (133–3041)	150 (20–600)	142 (17–558)	320 (60–1120)	321 (60–1118)	50 (10–210)	54 (8–206)
West Bengal	5650 (930–20 270)	638 (105–2291)	960 (120–3730)	108 (13–421)	2590 (500–8880)	331 (64–1133)	440 (60–1630)	56 (8–208)
**Regions**								
Central	37 170 (5850–135 840)	850 (134–3105)	6290 (750–24 700)	144 (17–565)	22 090 (3630–80 430)	540 (89–1967)	3740 (470–14 530)	91 (11–355)
East	22 310 (3620–80 490)	762 (124–2749)	3780 (460–14 800)	129 (16–506)	13 420 (2410–47 090)	437 (79–1534)	2270 (310–8620)	74 (10–281)
North	5700 (950–20 340)	596 (99–2127)	960 (120–3720)	101 (13–389)	2790 (560–9420)	282 (56–952)	470 (70–1760)	48 (7–177)
Northeast	2810 (480–10 020)	564 (97–2010)	480 (60–1810)	95 (12–363)	1670 (350–5490)	317 (67–1040)	280 (40–1020)	54 (8–193)
South	10 830 (2000–37 230)	476 (88–1636)	1830 (250–6840)	81 (11–301)	5180 (1140–16 750)	251 (55–810)	880 (140–3100)	42 (7–150)
West	10 040 (1730–35 380)	580 (100–2044)	1700 (220–6520)	98 (13–377)	7370 (1270–26 360)	453 (78–1619)	1250 (160–4780)	77 (10–294)
National†	83 830 (14 000–300 800)	657 (110–2357)	14 190 (1790–54 730)	111 (14–429)	49 800 (9100–174 180)	403 (74–1408)	8430 (1170–31 730)	68 (9–257)

Incidence rates are cases per 1000 children younger than 5 years. UI=uncertainty interval.

* Data are in thousands.

† Since state and region estimates are related to the total estimate only through the estimated rate in the unexposed group (ie, children without pneumonia risk factors), the state counts will not sum to the national counts.

In 2015, there were an estimated 143 000 cases (95% UI 23 000–530 000) of pneumonia and 24 000 cases (3000–95 000) of severe pneumonia among children with HIV, and the incidence of pneumonia and severe pneumonia among children with HIV was highest in Uttar Pradesh (4673 cases of pneumonia per 1000 children with HIV [652–17 877] and 791 cases of severe pneumonia per 1000 children with HIV [85–3293]). Between 2000 and 2015, the incidence of pneumonia and severe pneumonia in children with HIV increased in Rajasthan, Jharkhand, Bihar, Odisha, Jammu and Kashmir, and Haryana (appendix pp 18–19).

In 2015, the estimated national pneumonia case fatality rate was 0·38% (95% UI 0·11–2·10) and the estimated severe pneumonia case fatality rate was 2·26% (0·60–16·30). In 2015, pneumonia case fatality rates ranged from 0·87% (0·26–4·37) in Assam to 0·12% (0·03–0·69) in Maharashtra. Severe pneumonia case fatality rates ranged from 5·20% (1·40–36·42) in Assam to 0·72% (0·19–5·31) in Maharashtra (appendix p 17). Pneumonia mortality decreased in all states between 2000 and 2015.

**Figure 2:**
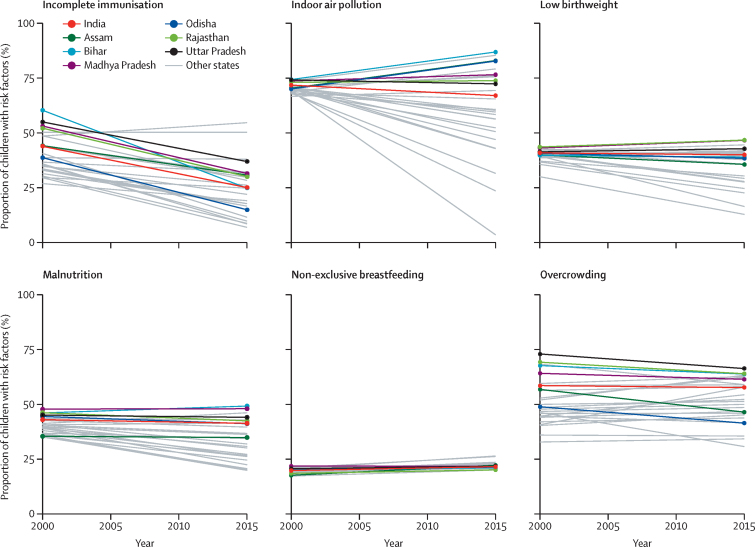
Prevalence of risk factors for pneumonia in India and high-focus* states (2000–15) States with high infant mortality are designated as high-focus states by the Government of India. *Risk factor prevalence data for Chhattisgarh, Jharkhand, and Uttarakhand in 2000 are unavailable as they were part of Madhya Pradesh, Bihar, and Uttar Pradesh, respectively, until late 2000.

The national prevalence of all seven pneumonia risk factors, with the exception of non-exclusive breastfeeding, decreased between 2000 and 2015 (figure 2). The prevalence of non-exclusive breastfeeding increased from 20% to 22% between 2000 and 2015. The prevalence of HIV infection was estimated to be the lowest of all pneumonia risk factors (0·3% in both 2000 and 2015). National prevalence of malnutrition decreased marginally; however, the prevalence of malnutrition in many of the high-focus states largely remained constant or increased, with the exception of Rajasthan and Odisha, in which the malnutrition prevalence decreased by 8% and 7%, respectively. The prevalence of incomplete immunisation decreased more than the other six pneumonia risk factors from 25% to 19% between 2000 and 2015. The range of prevalence estimates for indoor air pollution at the state level increased substantially between 2000 and 2015.

## Discussion

We calculated national-level and state-level estimates of pneumonia and severe pneumonia morbidity for India between 2000 and 2015 using an updated risk factor-based approach. To our knowledge, these estimates represent the first subnational pneumonia morbidity estimates and the most contemporary severe pneumonia morbidity estimates for India. Together with pneumonia mortality estimates prepared by the WHO/MCEE collaboration,^1^ these estimates can be used by policy makers to track progress toward reducing the burden of pneumonia and prioritising the introduction of new child health interventions. Furthermore, these estimates could be used to estimate the cost-effectiveness of pneumonia interventions.

We estimate that pneumonia and severe pneumonia cases in India decreased by approximately 41% between 2000 and 2015, which is greater than the 22% reduction in pneumonia cases observed globally during the same period.^12^ The reduction in India corresponds to an overall reduction in the proportion of children who are not immunised and state-specific reductions in low birthweight, malnutrition, and exposure to indoor air pollution. Many risk factors for pneumonia are associated with low socioeconomic status. Although considerable socioeconomic inequities remain in India, overall improvements in socioeconomic status would have contributed to a reduction in the prevalence of important risk factors for pneumonia.

Additionally, the launch of the National Rural Health Mission in 2005 (now the National Health Mission) has helped to expand and improve access to many primary health services for women and children in the country, including immunisation.^21^, ^22^ Researchers have also found that nutrition outcomes have been improving in several states in India at least partly due to improved infant and young child feeding practices encouraged by the Integrated Child Development Services programme.^23^ Additional government efforts aimed at addressing the prevalence of risk factors for pneumonia morbidity, including Mission Indradhanush to improve full immunisation coverage and Pradhan Mantri Ujjwala Yojana to extend access to liquid petroleum gas and therefore reduce exposure to indoor air pollution, could help further reduce the burden of pneumonia morbidity in the country.

Despite these reductions, considerable disparities in pneumonia morbidity remain between states in India. These disparities arise largely as a result of substantial differences in the prevalence of risk factors for pneumonia. All states in India had introduced the Hib-containing pentavalent vaccine by December, 2015. However, the states with the lowest pneumonia burden (Kerala and Tamil Nadu) were the first to introduce the vaccine in 2012. Many high burden states did not introduce the vaccine until 2015. Uttar Pradesh, the state with the highest estimated incidence of pneumonia and severe pneumonia, introduced the pentavalent vaccine in December, 2015. Additionally, differences in care-seeking behaviours between states are also likely to have contributed to disparities in the case fatality ratios estimated using state-level estimates of pneumonia mortality for 2015 from the WHO/MCEE collaboration.^1^

Although the prevalence of pneumonia risk factors in many states declined between 2000 and 2015, in many states the prevalence of many risk factors increased or remained stable, and thus should be the target of strategies to address the burden of pneumonia in India. The strategy used for the introduction of pneumococcal conjugate vaccine in 2017, in Uttar Pradesh, Bihar, and Himachal Pradesh—some of the states with the highest pneumonia incidence—differed from the strategy used for Hib vaccine which was introduced first in Tamil Nadu and Kerala. The introduction of pneumococcal conjugate vaccine in Uttar Pradesh and Bihar was initiated in several districts in these states. In the future, district-level estimates of pneumonia burden will be helpful for policy making with regard to the introduction of new vaccines that target respiratory pathogens including respiratory syncytial virus and influenza.

The 2015 national estimates of pneumonia and severe pneumonia of approximately 49·8 million and 8·4 million cases, respectively, are marginally higher than previously published pneumonia and severe pneumonia estimates (45·0 million and 7·2 million cases, respectively).^12^ Our 2015 estimates allow for correlation of pneumonia risk factors (ie, the previous model assumed independence of risk factors). As such, we would expect higher estimates, assuming all other factors remain constant, in settings with high correlation between risk factors. Additionally, our 2015 severe pneumonia estimates are also greater than the national estimates published for 2010.^10^ At the state-level, the number and incidence of severe pneumonia cases also differ substantially—the current estimates are higher than those for 2010 for all states. Although the rank order of states by the number of severe pneumonia cases is similar between the two models, the rank order by incidence differs substantially. This suggests differences in the denominator used to calculate incidence rates. The previously published estimates used a summary estimate comprising radiography-confirmed pneumonia rather than WHO-defined severe pneumonia to calculate severe pneumonia cases. The incidence of radiography-confirmed pneumonia from the three studies comprising the summary estimate^24^, ^25^, ^26^ are lower than the severe pneumonia incidence.

In 2016, a prospective and cross-sectional study was done in India, which provided estimates of community- acquired pneumonia incidence for 2016 for Bihar and Uttar Pradesh.^27^ In two districts in Uttar Pradesh, the estimated incidence of community-acquired pneumonia was 87 cases and 177 cases per 1000 children. The estimated incidence in two districts in Bihar was 208 and 221 cases per 1000 children. In our study, the estimated incidence of pneumonia among children younger than 5 years was 524 cases per 1000 children in Uttar Pradesh and 495 cases per 1000 children in Bihar. The authors suggest that their estimates are likely to underestimate the true incidence of community-acquired pneumonia in the four districts of these two states. The authors also noted that gender biases associated with access to care and patients with pneumonia dying before reaching a health facility might have contributed to the underestimation of community-acquired pneumonia incidence in these two states.

We updated the methods of our previous model used to estimate pneumonia and severe pneumonia cases. First, our updated model includes seven risk factors for pneumonia instead of the five or six risk factors used in previous models.^9^, ^10^ Our earlier models also assumed that the risk factors were mutually exclusive (eg, a child with malnutrition did not also have low birthweight), with later models making the less stringent assumption that the risk factors were independent. The completeness of the India survey data, however, allowed us to directly measure the number of children with each combination of risk factors using individual-level survey data. Therefore, with the exception of HIV infection, we were able to build the risk factor model using the observed correlation between risk factors. For HIV, we assumed that within each state and combination of risk factors, the number of children with HIV was proportionally related to the proportion of children whose mothers had HIV.

We accounted for several sources of uncertainty in our estimates of pneumonia morbidity, including pneumonia incidence rates, the proportion of pneumonia cases that were severe, odds ratios for pneumonia risk factors, the proportion of children with pneumonia risk factors, and coverage of the pneumococcal conjugate vaccine and Hib vaccine in children. The wide UIs reported reflect the multiple sources of uncertainty in our model. However, these uncertainty estimates might be too conservative. For example, the upper UI for pneumonia cases in 2015 in India was estimated to be 174 million. This exceeds the total population in this age group of approximately 119 million children.^28^ Improved methods for estimating uncertainty from all potential sources of uncertainty, or preferably increased empirical data collection, could help to address this issue.

The post-hoc adjustment for immunisation with the Hib vaccine assumes homogeneous vaccine coverage within each state. However, established differences in vaccine coverage exist within states and based on demographic and socioeconomic indicators.^29^ Overlooking these disparities in immunisation coverage is likely to result in an overestimation of the impact of vaccination and an underestimation of the burden of pneumonia morbidity in each of the Indian states. Subnational pneumococcal conjugate vaccine coverage estimates from the private sector were published for 2012.^19^ However, we did not use these estimates in our model since the overall national coverage was reported to be less than 1% for 2012. Additionally, children who received pneumococcal conjugate vaccine through the private sector are likely to be at lower risk of pneumococcal disease compared with those who did not receive pneumococcal conjugate vaccine in the private sector.

Our model accounts for the prevalence of indoor air pollution as an independent risk factor for pneumonia. However, we were unable to account for the prevalence of exposure to ambient air pollution, which has been observed to be associated with short-term and long-term increased risk of pneumonia in children.^30^ Considering the high prevalence of ambient air pollution exposure in India, this omission could lead to an underestimation in our pneumonia morbidity estimates. Additionally, we were unable to account for the increased risk associated with having a family member with an upper or lower respiratory infection, which are known risk factors for pneumonia in India.^31^, ^32^

India has made substantial progress toward reducing childhood pneumonia mortality in the past 15 years. The country has the largest population of children younger than 5 years worldwide. This cohort of children will soon become the largest working population in the world. Ensuring that these children are able to live healthy, productive lives will require the scale-up of a comprehensive package of important interventions that protect against and prevent pneumonia, including breastfeeding for the first 6 months of life, adequate nutrition, immunisation with pertussis, measles, Hib, and pneumococcal conjugate vaccine, and interventions that reduce exposure to air pollution. Increasing utilisation of these interventions will protect many of the children most at risk for pneumonia and could help prevent long-term sequelae, including restrictive lung disease, obstructive lung disease, and asthma.^33^ These interventions are crucial for the future health and economic prosperity in India.
