# Supplementary material for: National, regional, and state-level pneumonia and severe pneumonia morbidity in children in India: modelled estimates for 2000 and 2015
Source: Lancet Child Adolesc Health. 2020 Sep;4(9):678–87. doi: 10.1016/S2352-4642(20)30129-2 (PMC7457699; doi:10.1016/S2352-4642(20)30129-2)
Supplement: Supplementary appendix [file mmc1.pdf]

THE LANCET
Child & Adolescent Health

Supplementary appendix

This appendix formed part of the original submission and has been peer reviewed. We post it as supplied by the authors.

Supplement to: Wahl B, Knoll MD, Shet A, et al. National, regional, and state-level pneumonia and severe pneumonia morbidity in children in India: modelled estimates for 2000 and 2015. *Lancet Child Adolesc Health* 2019; **4:** 678–87.

Change in national, regional, and state-level pneumonia and severe pneumonia morbidity in India: modelled estimates for 2000 and 2015

Authors: Brian Wahl PhD,[1] Maria Deloria Knoll PhD,[1] Anita Shet MD,[1] Prof Madhu Gupta MD,[2] Prof Rajesh Kumar MD,[2] Li Liu PhD,[3,4] Yue Chu MSPH,[5] Molly Sauer MPH,[1] Prof Katherine L O'Brien,[1*] Prof Mathuram Santosham MD,[1] Prof Robert Black,[3] Prof Harry Campbell MD,[5] Prof Harish Nair PhD,[5,6] David A McAllister MD[7]

[1] International Vaccine Access Center (IVAC), Department of International Health, Johns Hopkins Bloomberg School of Public Health, Baltimore, Maryland, USA
[2] School of Public Health, Post Graduate Institute of Medical Education and Research, Chandigarh, India
[3] Institute for International Programs, Department of International Health, Johns Hopkins Bloomberg School of Public Health, Baltimore, Maryland, USA
[4] Department of Population, Family and Reproductive Health, Johns Hopkins Bloomberg School of Public Health, Baltimore, Maryland, USA
[5] Institute for Population Research, Department of Sociology, the Ohio State University, Columbus, Ohio, USA
[6] Centre for Global Health Research, Usher Institute of Population Health Sciences and Informatics, Medical School, University of Edinburgh, Edinburgh, Scotland, UK
[7] Public Health Foundation of India, New Delhi, India
[8] Institute of Health and Wellbeing, University of Glasgow, Glasgow, Scotland, UK

[*] Current affiliation: World Health Organization, Geneva, Switzerland

Correspondence: Brian Wahl, International Vaccine Access Center (IVAC), Department of International Health, Johns Hopkins Bloomberg School of Public Health, 415 N Washington Street, Floor 5, Baltimore, MD 21231, USA, bwahl@jhu.edu

1. GATHER checklist

GATHER checklist item	Section(s) or sources providing information
Objectives and funding	
1. Define the indicators, populations, and time periods for which estimates were made.	Indicators, populations, and time periods provided in methods section.
2. List the funding sources for the work.	Funding source provided in summary section.
Data inputs	
3. Describe how the data were identified and how the data were accessed.	Data identification and collection approaches provided in methods section and in previous publications.[1]
4. Specify the inclusion and exclusion criteria. Identify all ad-hoc exclusions.	Inclusion and exclusion criteria provided in appendix (p 3) and in previous publications.[1]
5. Provide information about all included data sources and their main characteristics. For each data source used, report reference information or contact name/institution, population represented, data collection method, years of data collection, sex and age range, diagnostic criteria or measurement method, and sample size, as relevant.	Data sources and characteristics provided in appendix (pp 6-14).
6. Identify and describe any categories of input data that have potentially important biases (e.g., based on characteristics listed in item 5).	Potentially important biases provided in discussion.
7. Describe and give sources for any other data inputs.	Other sources of data described in methods.
8. Provide all data inputs in a file format from which data can be efficiently extracted, including all relevant meta-data listed in item 5. For any data inputs that cannot be shared because of ethical or legal reasons provide a contact name or the name of the institution that retains the right to the data.	Data inputs available in Excel spreadsheet in online open access database when appropriate.
Data analysis	
9. Provide a conceptual overview of the data analysis method. A diagram may be helpful.	Conceptual model provided in previous publication.[1]
10. Provide a detailed description of all steps of the analysis, including mathematical formulae. This description should cover, as relevant, data cleaning, data pre-processing, data adjustments and weighting of data sources, and mathematical or statistical models.	Detailed description of analysis provided in methods, appendix, and previous publications.[1]
11. Describe how candidate models were evaluated and how the final models were selected.	Discussion of candidate model considerations provided in methods and discussed in detail in previous publication.[1]
12. Provide the results of an evaluation of model performance, if done, as well as the results of any relevant sensitivity analysis.	Model performance addressed in discussion section.
13. Describe methods of calculating uncertainty of the estimates. State which sources of uncertainty were, and were not, accounted for in the uncertainty analysis.	Uncertainty methods and sources provided in methods.
14. State how analytic or statistical source code used to generate estimates can be accessed.	Access to source code provided in online GitHub repository.
Results and discussion	
15. Provide published estimates in a file format from which data can be efficiently extracted.	Results in Excel spreadsheet provided in online GitHub repository.
16. Report a quantitative measure of the uncertainty of the estimates.	Uncertainty intervals provided with all results in manuscript.
17. Interpret results in light of existing evidence. If updating a previous set of estimates, describe the reasons for changes in estimates.	Discussion of results relevant to existing research provided in research in context, results, and discussion.
18. Discuss limitations of the estimates. Include a discussion of any modelling assumptions or data limitations that affect interpretation of the estimates.	Limitations of data and models provided in discussion.

2: Methods for estimating state-level, all-cause clinical and severe pneumonia cases

We used methods that have been previously described elsewhere to estimate the incidence of community-based pneumonia and severe pneumonia in low- and middle-income countries.[1] We treated states in India as countries in this model. The detailed methods for all developing countries, including subnational estimates in India, are described below.

Step 1

A systematic literature review was conducted to identify published data on the incidence of pneumonia from community-based longitudinal studies. From the literature, an estimate of the proportion of pneumonia cases that are severe was also estimated. Only studies that met the minimum quality criteria we included. These were: (1) community-based surveillance of a defined population of children had to have been carried out for a minimum period of one year and in multiples of 12 months because clinical pneumonia has such a markedly seasonal nature; (2) the study had to use a strategy of active case detection; and (3) case definitions had to be clearly defined and consistently applied.

Step 2

We conducted a systematic literature review to identify the risk factors for pneumonia in children aged 0-4 years. We selected risk factors which in meta-analyses had strong[2] and consistent statistically significant associations with pneumonia. These were:

- Malnutrition (weight for age <-2SD): odds ratio (OR) 4.5 (2.1-9.5)
- Low birth weight (<2500g): OR 3.6 (0.8-16.3)
- Non-exclusive breastfeeding (4 months): OR 2.7 (1.7-4.4)
- Indoor air pollution: OR 1.6 (1.1-2.3)
- HIV: OR 6.51 (5.86-7.24)
- Crowding (more than 5 persons per household): OR 1.9 (1.5-2.5)
- Incomplete immunisation (no measles immunisation at 12 months): OR 1.8 (1.3-2.5)

Step 3

We obtained data on the prevalence of all risk factors other than HIV from representative household surveys with large sample sizes—the demographic and health surveys (DHS). DHS surveys are generally carried out every 2 to 5 years and provide data on demography and health indicators. The state-level prevalence data (other than HIV) in each state were obtained from the National Family Health Survey (NFHS). The HIV data were obtained from the United Nations Programme on HIV/AIDS (UNAIDS) estimates for 2013. For states in India, the prevalence of HIV in children was estimated using the proportion of children in each state with mothers infected with HIV and the UNAIDS estimate of the odds ratio for HIV infection in children born to mothers infected with HIV. We ensured that the definitions of risk factors were the same in the studies estimating the risk ratios as in the surveys that measured prevalence of exposure to these risk factors.

Specifically, we defined each risk factor using one or more DHS/NFHS variables. Then, using individual patient data from the standard DHS/NFHS survey (excluding special surveys) for each state at the survey version closest to 2000 and 2015 respectively, we calculated the proportion of participants with each combination of risk factors.

Step 4

Apart from HIV, all variables were contained within the DHS survey for India, and so the joint distributions were observed. For HIV, we corrected the DHS-derived estimates of HIV to match the UNAIDS survey estimates for India. First, we calculated the odds ratio for the difference in maternal HIV for each child to the childhood HIV prevalence estimates at the whole-India level. Next, we applied this odds ratio to the state-level data to estimate the prevalence with HIV for each combination of the other risk factors.

Step 5

The overall incidence (of clinical pneumonia in community) and hospitalization rate (for physician diagnosed pneumonia) for developing countries and Indian states were obtained from the Poisson regression models as described elsewhere.

10,000 samples for each prediction were obtained. For the proportion of cases which were severe (i.e. with lower chest wall indrawing), the estimate from random effects meta-analysis (using Stata 11.1) was represented as a beta distribution, from which 10,000 samples were obtained using R. The incidence of severe pneumonia was calculated as the incidence of clinical pneumonia in the community times the proportion with severe pneumonia.

The overall incidence (of clinical and hospitalized pneumonia respectively) in a population is the sum of each stratum specific rate, weighted by the proportion in each stratum where there are j mutually exclusive strata:

$$Total\ rate = \frac{\sum x_j}{\sum n_j} = \sum_j \left(\frac{x_j}{n_j} \cdot \frac{n_0}{x_0} \cdot \frac{x_0}{n_0} \cdot \frac{n_j}{\sum n_j} \right)$$

where x is the number of cases in a stratum, n is the total population in a stratum and where $j = 0$ indicates the stratum which has no exposures (i.e., the unexposed) and $j = 1$ to $j = 127$ indicate the strata for each unique combination of risk factors. This equation can be re-written as:

$$Total\ rate = \frac{x_0}{n_0} \times \sum j \left(\frac{x_j}{n_j} \cdot \frac{n_0}{x_0} \cdot \frac{n_j}{\sum n_j} \right)$$

where $\frac{x_0}{n_0}$ is the rate in the unexposed, $\frac{x_j}{n_j} \cdot \frac{n_0}{x_0}$ is the rate ratio, and $\frac{n_j}{\sum n_j}$ is the proportion in each stratum.

Therefore, using the regional estimates (i.e., South East Asia) developing countries) of the total rate, the combined rate ratio for each stratum, and the proportion in each stratum, we can calculate the incidence in unexposed people for the South East Asia region. If we then assume that the rate in the unexposed is consistent within the country, we can then calculate the incidence rate for each Indian state:

$$Indian\ state\ specific\ rate = \frac{\sum x_j^s}{\sum n_j^s} = \frac{\sum x_j^r}{\sum n_j^r} \cdot \frac{\sum_j (\frac{x_j}{n_j} \cdot \frac{n_0}{x_0} \cdot \frac{n_j^r}{\sum n_j^r})}{\sum_j (\frac{x_j}{n_j} \cdot \frac{n_0}{x_0} \cdot \frac{n_j^s}{\sum n_j^s})}$$

where s indicates a Indian state-specific value and r indicates a regional value for South East Asia.

Step 6

Assumptions

- As with attributable fraction calculations, we assume that the pooled risk ratios obtained from a range of case-control/cohort studies in the South East Asia region were applicable across each Indian state. As described in Step 7 we added additional uncertainty to our estimates to relax this assumption somewhat. We assumed that odds ratios were the same as relative risks.
- As in our previous analysis for HIV and pneumonia, we assumed that there was an *n*-way interaction between each risk factor (i.e., 1.25-fold reduction) such that rate ratios combined sub-multiplicatively (e.g., for the group with malnutrition and low birth weight, $RR_{malnutrition}$ = 4.5, and $RR_{low\ birth\ weight}$ = 3.6, RR_{both} = (4.5 x 3.6) / 1.25 = 12.96 rather than 4.5 x 3.6 = 16.2; and for the group with malnutrition, low birth weight, and non-exclusive breastfeeding, $RR_{non\text{-}exclusive\ breast\ feeding}$ = 2.7, $RR_{all\ three}$ = (4.5 x 3.6 x 2.7)/1.25^2 = 27.99 rather than 4.5 x 3.6 x 2.7 = 43.74). None of the primary studies included in the meta-analysis reported interaction/heterogeneity of effect measure estimates on a multiplicative scale. However, although we noted that the majority of studies did not report testing for interaction (presumably because of an actual or perceived lack of statistical power) it seemed unlikely to us that all risks combined fully multiplicatively.

Therefore, we added a weak interaction term to shrink the combined rate ratios towards the null. This would have the effect of causing the state-specific estimates to be attenuated towards the overall estimate for the South East Asia region.

- In order to calculate the uncertainty for the proportion of the population in each Indian state within each of the j strata, we sampled from a Dirichlet distribution, a multivariate extension of the beta distribution. Using this approach, we did not need to assume independence of risk factors.
- Finally, we assumed that the rate among the unexposed (i.e., the risk in the absence of these seven risk factors) came from the same distribution for all Indian states. This is the same as assuming that there was no residual confounding.

Step 7

Using a simulation-based approach, we performed the calculation described in Step 5 to estimate the Indian state-specific rates along with uncertainty estimates as uncertainty ranges (UR). This was done by performing this calculation for each of the 10,000 samples from the distributions representing the incidence rates, disease severity proportion, odds ratios and risk factor proportions (methods used to obtain each of these were obtained as described above) within R. Full analysis code and data are available from the authors on request.

Step 7

In a similar manner to equation 3 within step 5, we calculated the incidence of pneumonia in HIV-uninfected children by summing those strata where children were HIV negative regardless of the presence/absence of other risk factors. Similarly, we calculated the incidence of pneumonia in HIV-infected children.

Step 8

Having obtained estimates of the total rate and rate in HIV-uninfected children in each Indian state, we used the standard formula for attributable fraction:

$$Attributable\ fraction = \frac{I_e - I_0}{I_e}$$

Or, equivalently:

$$Attributable\ fraction = \frac{RR\ - 1}{RR}$$

where I_e is the incidence in HIV-infected and I_0 is the incidence in HIV-uninfected in each Indian state; and RR is the risk ratio for pneumonia in HIV-infected (compared to HIV-uninfected). We then applied the attributable fraction to the rate among the HIV-infected (and the UR) in order to obtain the rate attributable to HIV with the UR. As we had already incorporated uncertainty in the HIV risk factor estimates in calculating the total state rates, we did not add additional uncertainty at this stage.

3. Details of studies reporting incidence of clinical pneumonia in children younger than 5 years

S.No	Location (study period)	Region	Duration of study (months)	Age group under study (months)	Case definition		Status of Hib vaccine implementation at time of study	Status of PCV implementation at time of study (indicate valency of the vaccine)	Assessor	Surveillance intervals	Site of diagnosis	Denominator (cohort size)	Incidence 0-4 years (episodes/child-year)	Proportion of cases with severe pneumonia
					Clinical pneumonia	Severe pneumonia								
**1**	Ibadan, Nigeria; urban (Oct 1984 - Oct 1987)[3]	AFR	36	0-59	IC, W-, CR+		No	No	FW (and Physician)	Weekly	Clinic and home	610	1.38	
**2**	Maragua, Kenya; rural (Feb 1985 - Jan 1988)[4]	Africa	36	0-59	IC, W-, CR+		No	No	FW (and Physician)	Weekly	Clinic and home	470	0.16	
**3**	Basse, The Gambia; rural (Mar 1987 - Mar 1988)[5]	Africa	12	0-59	IB, W-, CR-		No	No	TFW (and Physician)	Weekly	Home and hospital	491	0.45	
**4**	Accra, Ghana; rural (Jan 1987 - Dec 1989)[6]	Africa	36	0-59	IB, W-, CR-		No	No	Nurses (and Physician)	Weekly	Clinic and home	1350	0.06	
**5**	Ilorin, Nigeria; suburban (Jul 1988 - Jun 1989)[7]	Africa	12	0-59	IA, W+, CR-	IA, W+, CR+	No	No	TFW	3x/week	Home	481	1.65	0.11
**6**	Ibadan, Nigeria; urban and rural (Jun 1999 - May 2001)[8]	Africa	24	0-59	IB, W-, CR-		No	No	Nurses (TFW)	Weekly	Home and hospital	1579	0.27	

* I A–C = WHO definitions (A - WHO diagnosis only, B - with 1-3 additional criteria, C - with 4 or more additional criteria); II = Physician's assessment; III + CXR +; W+ = Wheeze part of case definition; W– = Wheeze not part of case definition; C+/R+ = Crepitations/rales part of definition of acute lower respiratory infection; C– /R– = Crepitations/rales not part of definition of acute lower respiratory infection

S.No	Location (study period)	Region	Duration of study (months)	Age group under study (months)	Case definition		Status of Hib vaccine implementation at time of study	Status of PCV implementation at time of study (indicate valency of the vaccine)	Assessor	Surveillance intervals	Site of diagnosis	Denominator (cohort size)	Incidence 0-4 years (episodes/child-year)	Proportion of cases with severe pneumonia
					Clinical pneumonia	Severe pneumonia								
**7**	Upper and Central River Division, The Gambia; rural (Aug 2000 - Apr 2004))[9]	Africa	45	3-29	IB, W-, CR-	II	No	No	Nurses (and Physician)	Weekly	Hospital	8151	0.2	0.07
**8**	Paarl, South Arica; periurban (May 2012 - May 2014)[10]	Africa	24	0-11	IA, W-, CR-	IA, W-, CR+	Yes	Yes	TFW (and Physician)	Not known	Clinic and hospital	697	0.22	0.23
**9**	Monte-video, Uruguay; urban (May 1985 - Dec 1987)[11]	Americas	32	0-35	II		No	No	Pediatrician	10 days	Home	166	2.26	
**10**	Guatemala city, Guatemala; suburban (Jan 1985 - Dec 1986)[12]	Americas	24	0-59	IC, W-, CR+		No	No	TFW (and Physician)	Fortnightly	Clinic	521	0.31	
**11**	Cali, Colombia; urban (Jan 1987 - Dec 1989)[13]	Americas	36	0-17	IC, W-, CR+		No	No	TFW	Weekly	Clinic	340	1.26	
**12**	San Marcos, Guatemala; rural (Oct 2002 - Dec 2004)[14]	Americas	15	0-59	II	II	No	No	Physician	Weekly	Home	253	0.28	0.34

S.No	Location (study period)	Region	Duration of study (months)	Age group under study (months)	Case definition		Status of Hib vaccine implementation at time of study	Status of PCV implementation at time of study (indicate valency of the vaccine)	Assessor	Surveillance intervals	Site of diagnosis	Denominator (cohort size)	Incidence 0-4 years (episodes/child-year)	Proportion of cases with severe pneumonia
					Clinical pneumonia	Severe pneumonia								
**13**	Canto Grande, Peru; suburban (Jul 1987 - Oct 1989)[15]	Americas	27	0-41	II		No	No	Physician (and TFW)	2x/week	Home and clinic	1500	0.26	
**14**	Ghizer, Pakistan; rural (Nov 2001 - Dec 2002)[16]	Eastern Mediterranean	14	2-35	IC, W-, CR-	IC, W-, CR+	No	No	TFW	Fortnightly	Home and hospital	5204	0.23	0.27
**15**	Karachi, Pakistan; urban (Apr 2002 - Apr 2003)[17]	Eastern Mediterranean	12	0-59	IA, W-, CR-		No	No	TFW	Weekly	Home	1634	0.9	
**16**	Haryana, India; rural (Jan 1982 - Sep 1983)[18]	South East Asia	21	0-23	IB, W+, CR-		No	No	TFW	Weekly	Home	347	0.24	
**17**	Kathmandu, Nepal; rural (Feb 1984 - Jan 1987)[19]	South East Asia	36	0-59	IB, W+, CR+		No	No	TFW	Fortnightly	Home	1019	0.22	
**18**	Bangkok, Thailand; urban (Jan 1986 - Dec 1987)[20]	South East Asia	24	0-59	IC, W-, CR+		No	No	TFW (and Physician)	2x/week	Home	674	0.07	
**19**	Haryana, India; rural (Jan 1986 - Dec 1986)[21]	South East Asia	12	0-59	IB, W-, CR+		No	No	TFW	Fortnightly	Home	5078	0.54	

S.No	Location (study period)	Region	Duration of study (months)	Age group under study (months)	Case definition		Status of Hib vaccine implementation at time of study	Status of PCV implementation at time of study (indicate valency of the vaccine)	Assessor	Surveillance intervals	Site of diagnosis	Denominator (cohort size)	Incidence 0-4 years (episodes/child-year)	Proportion of cases with severe pneumonia
					Clinical pneumonia	Severe pneumonia								
**20**	Yumla, Nepal; rural (Jun 1986 - Jun 1989)[22]	South East Asia	36	0-59	IA, W+, CR-		No	No	TFW	Fortnightly	Home	13404	0.7	
**21**	Matlab, Bangladesh; rural (May 1988 - Apr 1989)[23]	South East Asia	12	0-59	IB, W-, CR-		No	No	TFW	2x/week	Home	696	0.23	
**22**	Wardha, India; rural (Sep 1990 - Aug 1991)[24]	South East Asia	12	0-59	IA, W+, CR-		No	No	Not reported (probably TFW)	Fortnightly	Home	384	0.08	
**23**	Mirzapur, Bangladesh; rural (Oct 1993 - Sep 1996)[25]	South East Asia	36	0-23	IB, W+, CR-	IB, W+, CR+	No	No	TFW (and pediatrician)	2x/week	Home and hospital	288	0.38	0.26
**24**	Bandung, Indonesia; suburban and rural (Feb 1999 - Jan 2001)[7]	South East Asia	24	0-59	IC, W+, CR-	IC, W+, CR+	No	No	TFW	Weekly	Home and clinic	1420	0.19	0.12

S.No	Location (study period)	Region	Duration of study (months)	Age group under study (months)	Case definition		Status of Hib vaccine implementation at time of study	Status of PCV implementation at time of study (indicate valency of the vaccine)	Assessor	Surveillance intervals	Site of diagnosis	Denominator (cohort size)	Incidence 0-4 years (episodes/child-year)	Proportion of cases with severe pneumonia
					Clinical pneumonia	Severe pneumonia								
**25**	Ballabgarh, Haryana, India; rural (Oct 2001 - Mar 2005)[26]	South East Asia	42	0-35	IC, W-, CR-	IC, W-, CR+	No	No	TFW (and Physician)	Weekly	Home and hospital	281	0.24	0.17
**26**	Dhaka, Bangladesh; urban (Apr 2004 - Dec 2007)[27]	South East Asia	45	0-59	IC, W-, CR-	IC, W+, CR+	No	No	TFW (and Physician)	Weekly	Home and hospital	12062	0.47	0.070
**27**	Mirzapur, Bangladesh; rural (Jul 2004 - Jun 2007)[28]	South East Asia	36	0-59	IC, W+, CR-	IC, W+, CR+	No	No	TFW (and Physician)	Weekly	Home and hospital	22378	0.31	0.53
**28**	Delhi, India; periurban (Jan 2011 - Jan 2012)[29]	South East Asia	12	0-59	IA, W-, CR-	IA, W-, CR+	No	No	TFW (and Physician)	Fortnightly	Home and hospital	106	0.37	0.06
**29**	Tari & Asaro, Papua New Guinea; rural (Jan 1979 - Jan 1983)[30]	Western Pacific	48	0-59	IB, W-, CR-	IB, W-, CR+	No	No	TFW (and Physician)	Fortnightly	Home and hospital	1595	0.41	0.7
**30**	Changping county, China; semirural (Jun 1981 - Jun 1983)[32]	Western Pacific	24	0-143	II		No	No	Physician	Weekly	Home and primary school	526	0.07	

S.No	Location (study period)	Region	Duration of study (months)	Age group under study (months)	Case definition		Status of Hib vaccine implementation at time of study	Status of PCV implementation at time of study (indicate valency of the vaccine)	Assessor	Surveillance intervals	Site of diagnosis	Denominator (cohort size)	Incidence 0-4 years (episodes/child-year)	Proportion of cases with severe pneumonia
					Clinical pneumonia	Severe pneumonia								
**31**	Albang Manilla, Phillipines; urban (Apr 1985 - Mar 1987)[32]	Western Pacific	24	0-59	IC, W-, CR+		No	No	Nurse (and Physician)	Weekly	Home and clinic	1978	0.53	
**32**	Asaro valley, Papua New Guinea; rural (Jan 1985 - Dec 1987)[33]	Western Pacific	36	0-59	IB, W+, CR-		No	No	Lay reporters	2x/week	Home	156	1.32	
**33**	Zhejiang Province, China; rural (Apr 1990 - Mar 1991)[34]	Western Pacific	12	0-59	IA, W-, CR-		No	No	TFW (and Physician)	Fortnightly	Home and clinic	1215	0.10	
**34**	Zhejiang Province, China; rural (Apr 1990 - Mar 1991)[35]	Western Pacific	12	0-59	IA, W-, CR-		No	No	TFW (and Physician)	Monthly	Home and clinic	7472	0.13	
**35**	Rudong County, Jiangsu Province, China; rural (Feb 1991 - Jan 1993)[36]	Western Pacific	12	0-59	IA, W-, CR-		No	No	TFW (and Physician)	Not known	Home, clinic and hospital	10541	0.28	
**36**	Liyang City, China; rural (1991 - 1994)[37]	Western Pacific	36	0-59	IA, W-, CR-		No	No	TFW (and Physician)	Not known	Clinic and hospital	20867	0.12	

S.No	Location (study period)	Region	Duration of study (months)	Age group under study (months)	Case definition		Status of Hib vaccine implementation at time of study	Status of PCV implementation at time of study (indicate valency of the vaccine)	Assessor	Surveillance intervals	Site of diagnosis	Denominator (cohort size)	Incidence 0-4 years (episodes/child-year)	Proportion of cases with severe pneumonia
					Clinical pneumonia	Severe pneumonia								
**37**	Changshou County, China; rural (Feb 1992 - Jan 1993)[38]	Western Pacific	12	0-59	IA, W-, CR-		No	No	TFW	Not known	Clinic	2246	0.19	
**38**	Huanan and Keshan County, Heilongjiang Province; rural (Jan 1993 - Dec 1993)[39]	Western Pacific	12	0-59	IA, W-, CR-		No	No	TFW	Not known	Clinic	5812	0.16	
**39**	Guangzhou, China; urban (Oct 1993 - Sep 1998)[40]	Western Pacific	60	0-59	IC, III		No	No	TFW	Not known	Clinic	120970	0.32	
**40**	Liyang City, China; rural (Feb 1994 - Jan 1995)[41]	Western Pacific	12	0-59	IA, W-, CR-		No	No	TFW (and Physician)	Not known	Clinic and hospital	11729	0.12	
**41**	Henan Province, China; rural and urban (Jan 1994 - Dec 1994)[42]	Western Pacific	12	0-59	IA, W-, CR-		No	No	TFW (and Physician)	Not known	Clinic and hospital	7917	0.11	
**42**	Licheng District, Quanzhou City, China; rural (Oct 1994 - Sept 1995)[43]	Western Pacific	12	0-59	IA, W-, CR-		No	No	TFW (and Physician)	Not known	Clinic and hospital	4665	0.13	

S.No	Location (study period)	Region	Duration of study (months)	Age group under study (months)	Case definition		Status of Hib vaccine implementation at time of study	Status of PCV implementation at time of study (indicate valency of the vaccine)	Assessor	Surveillance intervals	Site of diagnosis	Denominator (cohort size)	Incidence 0-4 years (episodes/child-year)	Proportion of cases with severe pneumonia
					Clinical pneumonia	Severe pneumonia								
**43**	Huaning County, Yunnan Province, China (Jan 1995 - Dec 1997)[44]	Western Pacific	24	0-59	IA, W-, CR-		No	No	TFW (and Physician)	Not known	Clinic and hospital	6966	0.66	
**44**	Hubei Province, China; rural (Jul 1996- Jun 1999)[45]	Western Pacific	36	0-59	IA, W-, CR-		No	No	TFW	Not known	Clinic	75337	0.64	
**45**	Jinan City, China; urban (Jan 1995- Dec 2001)[46]	Western Pacific	84	0-59	II		No	No	TFW	Not known	Clinic	321249	0.22	
**46**	Haryana, India; rural (Aug 2012- Aug 2013)[47]	South East Asia	12	0-119	IB, W-, CR-		No	No	TFW	Weekly	Home and hospital	1782	0.37	

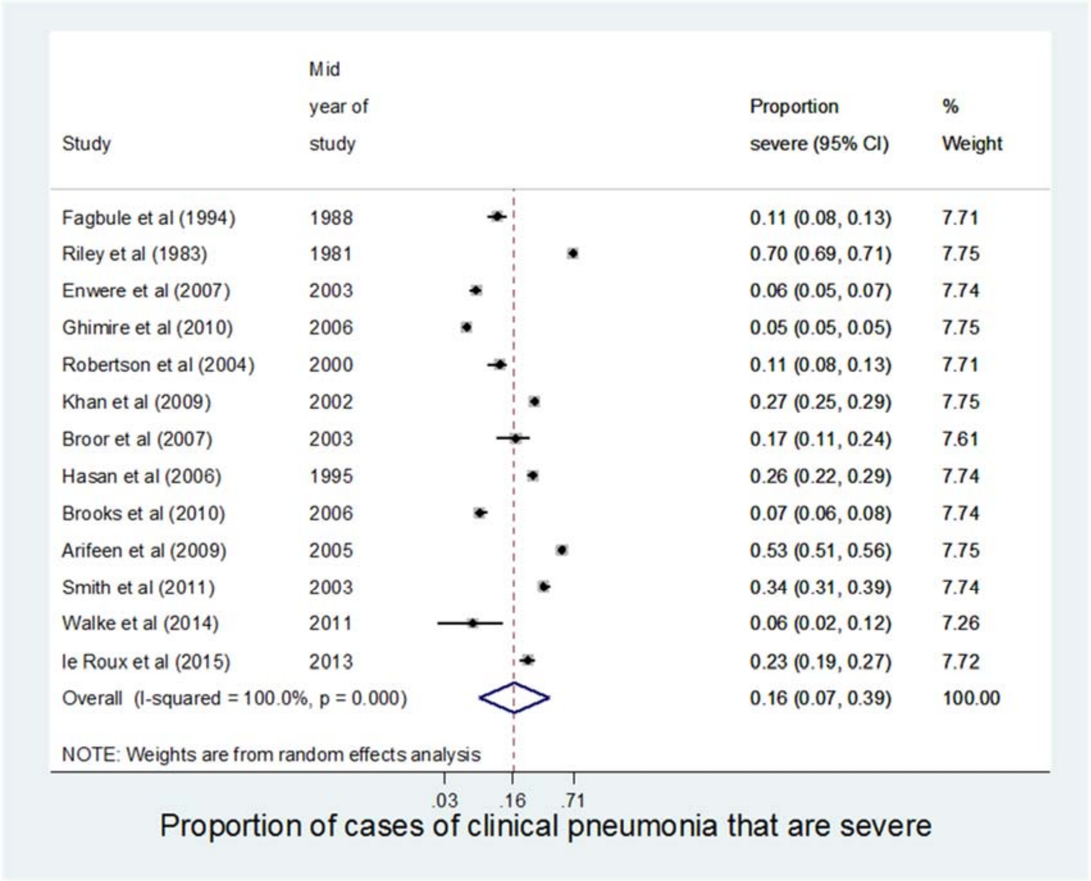

Studies reporting the proportion of children with clinical pneumonia having severe pneumonia (lower chest wall indrawing)

4: State-level pneumonia risk factor prevalence (National Family Health Survey)

State	Year	HIV	Incomplete immunization	Exposure to indoor air pollution	Low birth weight	Malnutrition	Non-exclusive breast feeding	Over-crowding
Andaman & Nicobar Islands	2000	-	-	-	-	-	-	-
Andaman & Nicobar Islands	2015	0.62	34.78	50.93	40.06	29.50	27.33	46.89
Andhra Pradesh	2000	0.68	32.99	70.15	40.65	40.56	17.77	46.77
Andhra Pradesh	2015	0.19	16.66	42.87	38.30	36.80	22.44	48.59
Arunachal Pradesh	2000	0.82	48.57	67.76	40.41	39.59	21.02	42.04
Arunachal Pradesh	2015	0.34	54.65	60.71	27.85	26.22	26.20	43.82
Assam	2000	0.30	44.17	70.39	39.98	35.49	17.65	56.83
Assam	2015	0.37	30.83	83.00	35.65	34.88	22.19	46.46
Bihar	2000	0.16	60.29	74.38	39.75	46.12	20.03	67.70
Bihar	2015	0.38	24.85	86.92	38.98	49.25	21.06	63.84
Chandigarh	2000	-	-	-	-	-	-	-
Chandigarh	2015	1.55	7.22	12.37	43.30	28.87	21.13	45.88
Chhattisgarh	2000	-	-	-	-	-	-	-
Chhattisgarh	2015	0.24	12.96	78.69	38.93	42.88	19.42	52.92
Dadra & Nagar Haveli	2000	-	-	-	-	-	-	-
Dadra & Nagar Haveli	2015	0.93	20.50	53.11	50.62	42.86	25.47	46.89
Daman & Diu	2000	-	-	-	-	-	-	-
Daman & Diu	2015	0.25	24.32	27.52	34.40	33.17	20.64	46.44
Delhi	2000	0.62	33.62	69.22	39.06	40.91	20.77	48.58
Delhi	2015	0.51	16.65	3.54	41.90	31.96	20.63	52.28
Goa	2000	-	35.38	68.46	42.56	36.15	18.97	44.62
Goa	2015	0.48	9.86	23.56	41.59	26.68	20.91	41.59
Gujarat	2000	0.16	36.68	68.34	40.59	41.31	21.53	51.99
Gujarat	2015	0.41	32.17	65.51	41.22	46.18	22.29	63.32
Haryana	2000	0.31	34.73	69.46	39.84	36.87	18.69	59.55
Haryana	2015	0.36	26.21	59.55	40.54	34.48	21.71	62.70
Himachal Pradesh	2000	0.34	31.46	66.70	40.96	38.10	21.62	40.73
Himachal Pradesh	2015	0.14	17.79	69.38	40.76	27.21	20.79	57.90
India	2000	0.35	43.87	71.80	40.89	43.05	19.81	58.60
India	2015	0.34	25.12	67.04	40.05	41.39	21.56	57.66
Jammu & Kashmir	2000	0.11	33.33	70.48	41.86	39.17	19.64	68.35
Jammu & Kashmir	2015	0.23	17.79	58.40	37.80	22.47	21.37	59.05
Jharkhand	2000	-	-	-	-	-	-	-
Jharkhand	2015	0.33	20.77	71.30	39.39	52.05	20.68	58.49
Karnataka	2000	0.58	35.05	70.69	40.51	40.73	19.49	58.69
Karnataka	2015	0.42	22.02	56.40	38.23	41.49	23.02	58.93
Kerala	2000	0.57	30.36	70.33	36.77	35.17	18.90	44.10

Kerala	2015	0.20	8.81	50.61	24.65	20.71	21.45	46.79
Lakshadweep	2000	-	-	-	-	-	-	-
Lakshadweep	2015	0.65	6.17	70.78	29.55	27.60	17.86	78.90
Madhya Pradesh	2000	0.40	53.11	73.72	43.12	47.88	21.88	64.17
Madhya Pradesh	2015	0.35	31.55	76.56	46.64	48.04	21.69	61.46
Maharashtra	2000	0.23	29.69	70.77	41.83	43.61	18.41	52.88
Maharashtra	2015	0.36	24.86	56.29	42.61	42.89	21.73	62.33
Manipur	2000	0.60	43.82	70.72	35.66	35.26	18.92	47.81
Manipur	2015	0.30	29.79	60.31	22.64	19.94	23.03	50.02
Meghalaya	2000	0.99	48.64	73.70	39.95	39.95	19.35	44.67
Meghalaya	2015	0.29	28.37	85.39	27.58	36.29	22.14	54.39
Mizoram	2000	0.23	38.73	69.95	30.05	35.92	19.72	50.00
Mizoram	2015	0.35	38.00	46.99	12.82	20.41	23.59	51.36
Nagaland	2000	0.27	50.27	68.72	39.57	35.03	20.59	40.37
Nagaland	2015	0.30	50.27	79.21	29.24	24.61	26.44	45.21
Odisha	2000	0.43	38.71	70.07	40.71	44.50	20.57	48.93
Odisha	2015	0.32	15.01	82.79	38.41	41.23	21.38	41.43
Puducherry	2000	-	-	-	-	-	-	-
Puducherry	2015	0.46	8.42	15.73	26.09	25.81	24.33	45.61
Punjab	2000	0.35	29.58	71.23	41.76	36.66	17.29	56.38
Punjab	2015	0.36	7.00	43.00	44.56	26.25	20.09	58.13
Rajasthan	2000	0.28	52.15	73.08	43.59	46.20	18.52	69.26
Rajasthan	2015	0.35	30.11	73.88	46.74	42.50	20.12	63.99
Sikkim	2000	0.25	35.88	68.70	39.44	35.11	18.58	46.31
Sikkim	2015	0.30	8.56	52.34	16.42	20.70	22.79	30.75
Tamil Nadu	2000	0.27	26.92	68.17	37.14	39.92	20.09	32.82
Tamil Nadu	2015	0.45	19.14	31.56	30.37	30.14	23.14	34.26
Telangana	2000	-	-	-	-	-	-	-
Telangana	2015	0.54	19.90	38.94	36.55	35.19	21.63	38.20
Tripura	2000	0.53	44.00	71.20	38.67	38.93	19.73	36.00
Tripura	2015	0.30	31.58	75.64	39.55	30.83	23.01	35.56
Uttar Pradesh	2000	0.30	54.99	74.20	41.36	45.12	20.65	73.01
Uttar Pradesh	2015	0.30	36.96	72.39	42.82	44.12	21.76	66.36
Uttarakhand	2015	0.36	22.28	62.11	43.85	32.41	21.30	51.86
West Bengal	2000	0.53	40.63	70.81	41.23	42.68	20.43	45.88
West Bengal	2015	0.38	11.62	76.13	35.77	39.66	20.89	45.96

5: State-specific pneumonia and severe pneumonia case fatality for select states

State	Y ear	Pneumonia CFR (UI)	Severe pneumonia CFR (UI)
Andhra Pradesh	2015	0.40% (0.12 - 1.95%)	2.38% (0.66 - 14.78%)
Assam	2015	0.87% (0.26 - 4.37%)	5.20% (1.40 - 36.42%)
Bihar	2015	0.34% (0.10 - 1.94%)	2.02% (0.53 - 15.66%)
Chhattisgarh	2015	0.45% (0.13 - 2.50%)	2.63% (0.69 - 16.67%)
Delhi	2015	0.21% (0.06 - 1.14%)	1.31% (0.33 - 9.15%)
Gujarat	2015	0.28% (0.08 - 1.66%)	1.66% (0.42 - 13.84%)
Haryana	2015	0.39% (0.11 - 1.94%)	2.36% (0.62 - 16.49%)
Himachal Pradesh	2015	0.32% (0.09 - 1.53%)	2.04% (0.51 - 15.28%)
India	2015	0.38% (0.11 - 2.00%)	2.26% (0.60 - 16.3%)
Jammu & Kashmir	2015	0.24% (0.08 - 1.06%)	1.36% (0.41 - 9.50%)
Jharkhand	2015	0.26% (0.07 - 1.48%)	1.57% (0.41 - 12.58%)
Karnataka	2015	0.20% (0.06 - 1.05%)	1.18% (0.32 - 8.01%)
Kerala	2015	0.26% (0.10 - 0.92%)	1.53% (0.51 - 9.16%)
Madhya Pradesh	2015	0.47% (0.13 - 3.01%)	2.78% (0.71 - 24.10%)
Maharashtra	2015	0.12% (0.03 - 0.69%)	0.72% (0.19 - 5.31%)
Odisha	2015	0.49% (0.14 - 2.78%)	2.88% (0.76 - 19.46%)
Punjab	2015	0.32% (0.09 - 1.60%)	1.93% (0.51 - 19.25%)
Rajasthan	2015	0.36% (0.10 - 2.23%)	2.16% (0.55 - 18.08%)
Tamil Nadu	2015	0.28% (0.10 - 1.07%)	1.63% (0.51 - 9.26%)
Uttar Pradesh	2015	0.43% (0.12 - 2.59%)	2.56% (0.66 - 19.88%)
Uttarakhand	2015	0.35% (0.10 - 1.87%)	2.24% (0.53 - 11.20%)
West Bengal	2015	0.29% (0.08 - 1.49%)	1.7% (0.46 - 12.44%)

5: National and state-level estimates of pneumonia and severe pneumonia morbidity among children less than five years infected with HIV

	2000				2015			
**Geography**	**Pneumonia cases in thousands (UI)**	**Pneumonia incidence (UI)**	**Severe pneumonia cases in thousands (UI)**	**Severe pneumonia incidence (UI)**	**Pneumonia cases in thousands (UI)**	**Pneumonia incidence (UI)**	**Severe pneumonia cases in thousands (UI)**	**Severe pneumonia incidence (UI)**
**State**								
Andhra Pradesh	9 (2-24)	2450 (588-6783)	1 (0-4)	416 (74-1284)	6 (1-23)	2261 (357-8585)	1 (0-4)	382 (46-1566)
Assam	2 (0-6)	1127 (217-4098)	0 (0-1)	191 (28-775)	4 (1-13)	2483 (396-9275)	1 (0-2)	421 (51-1680)
Bihar	32 (6-97)	5610 (1035-17272)	5 (1-18)	951 (136-3254)	18 (3-65)	3143 (555-11138)	3 (0-12)	533 (71-2022)
Chhattisgarh	6 (1-27)	5062 (559-21605)	1 (0-5)	858 (73-3829)	4 (1-16)	3682 (475-14497)	1 (0-3)	623 (62-2585)
Delhi	1 (0-4)	2018 (449-5816)	0 (0-1)	342 (59-1110)	1 (0-5)	2361 (339-9112)	0 (0-1)	400 (44-1656)
Goa	0 (0-0)	0 (0-0)	0 (0-0)	0 (0-0)	0 (0-1)	3965 (350-16894)	0 (0-0)	673 (47-3044)
Gujarat	14 (1-60)	5230 (478-21966)	2 (0-11)	885 (64-3897)	11 (1-46)	4665 (563-19639)	2 (0-8)	784 (73-3391)
Haryana	3 (1-10)	2595 (509-8326)	1 (0-2)	441 (66-1570)	4 (0-15)	3492 (446-13971)	1 (0-3)	592 (59-2499)
Himachal Pradesh	2 (0-8)	6158 (512-26593)	0 (0-1)	1046 (69-4846)	0 (0-2)	1764 (249-7236)	0 (0-0)	301 (32-1278)
Jammu and Kashmir	0 (0-1)	607 (189-1386)	0 (0-0)	103 (23-277)	1 (0-3)	1388 (254-4915)	0 (0-1)	236 (32-928)
Jharkhand	9 (2-29)	5610 (1035-17272)	2 (0-5)	951 (136-3254)	5 (1-19)	3316 (565-11734)	1 (0-3)	562 (73-2138)
Karnataka	11 (2-40)	4339 (641-15714)	2 (0-7)	733 (85-2897)	4 (1-12)	1623 (307-5663)	1 (0-2)	276 (40-1029)
Kerala	3 (0-11)	1929 (270-7465)	0 (0-2)	326 (36-1340)	1 (0-2)	652 (171-1755)	0 (0-0)	110 (22-329)
Madhya Pradesh	19 (2-82)	5062 (559-21605)	3 (0-15)	858 (73-3829)	13 (2-50)	4009 (601-15554)	2 (0-9)	679 (79-2764)
Maharashtra	13 (2-50)	2646 (412-10112)	2 (0-9)	446 (53-1839)	12 (2-42)	2948 (530-10535)	2 (0-8)	496 (69-1910)
Northeast	0 (0-10)	3857 (468-15480)	0 (0-0)	645 (63-2785)	0 (0-10)	3921 (487-16030)	0 (0-0)	658 (64-2860)
Odisha	4 (1-12)	2206 (457-6635)	1 (0-2)	374 (59-1232)	3 (1-11)	2196 (450-7130)	1 (0-2)	373 (58-1303)
Punjab	0 (0-1)	396 (131-880)	0 (0-0)	67 (16-176)	2 (0-8)	2231 (343-8673)	0 (0-1)	379 (44-1558)
Rajasthan	21 (3-75)	5732 (928-20035)	4 (0-14)	973 (119-3638)	15 (2-59)	4620 (565-18713)	2 (0-11)	782 (74-3351)
Tamil Nadu	16 (2-69)	6144 (620-26255)	3 (0-13)	1038 (82-4773)	5 (1-19)	2128 (288-8350)	1 (0-3)	360 (38-1507)

Union territories	0 (0-0)	3535 (445-14162)	0 (0-0)	591 (59-2559)	0 (0-0)	1521 (330-4911)	0 (0-0)	256 (43-911)
Uttar Pradesh	145 (14-622)	13463 (1299-57862)	25 (2-111)	2284 (174-10353)	41 (6-156)	4673 (652-17877)	7 (1-29)	791 (85-3293)
Uttarakhand	6 (1-28)	13463 (1299-57862)	1 (0-5)	2284 (174-10353)	1 (0-4)	2257 (293-9372)	0 (0-1)	382 (38-1667)
West Bengal	9 (2-30)	2156 (397-7688)	1 (0-6)	366 (51-1412)	7 (1-26)	2115 (314-8534)	1 (0-5)	356 (40-1490)
**Region**								
Central	190 (20-820)	9927 (1066-41706)	30 (0-150)	1660 (146-7476)	70 (10-280)	4483 (614-17142)	10 (0-50)	753 (80-3088)
East	50 (10-160)	4127 (856-12378)	10 (0-30)	690 (111-2293)	30 (10-120)	2789 (483-9852)	10 (0-20)	469 (63-1792)
North	10 (0-50)	3181 (552-11399)	0 (0-10)	532 (72-2052)	10 (0-40)	2417 (369-8971)	0 (0-10)	407 (47-1626)
Northeast	0 (0-20)	1951 (322-7392)	0 (0-0)	326 (42-1326)	10 (0-20)	2953 (429-11373)	0 (0-0)	497 (56-2022)
South	40 (10-140)	3861 (651-13492)	10 (0-30)	646 (84-2486)	20 (0-50)	1854 (315-6600)	0 (0-10)	312 (41-1209)
West	30 (0-110)	3579 (478-13997)	0 (0-20)	599 (62-2514)	20 (0-90)	3590 (557-13331)	0 (0-20)	605 (73-2440)
**National**								
India	222 (37-797)	3883 (642-13948)	38 (5-145)	657 (83-2545)	143 (23-530)	2914 (460-10835)	24 (3-95)	493 (59-1935)

References

1. McAllister DA, Liu L, Shi T, et al. Global, regional, and national estimates of pneumonia morbidity and mortality in children younger than 5 years between 2000 and 2015: a systematic analysis. The Lancet Global Health 2018.
2. Craun GF, Calderon RL. How to interpret epidemiological associations. Nutrients in Drinking Water 2005; 108: 108-15.
3. Oyejide C, Osinusi K. Acute respiratory tract infection in children in Idikan community, Ibadan, Nigeria: severity, risk factors, and frequency of occurrence. *Review of Infectious Diseases* 1990; **12**(Supplement 8): S1042-SI046.
4. Wafula E, Onyango F, Mirza W, et al. Epidemiology of acute respiratory tract infections among young children in Kenya. *Review of infectious diseases* 1990; **12**(Supplement 8): S1035-S8.
5. Campbell H, Lamont A, O'Neill K, et al. Assessment of clinical criteria for identification of severe acute lower respiratory tract infections in children. *The Lancet* 1989; **333**(8633): 297-9.
6. Afari E, SAKATOKU H, NAKANO T, et al. Acute respiratory infections in children under five in two rural communities in southern Ghana. *Japanese Journal of Tropical Medicine and Hygiene* 1991; **19**(3): 275-80.
7. Fagbule D, Parakoyi D, Spiegel R. Acute respiratory infections in Nigerian children: prospective cohort study of incidence and case management. *Journal of Tropical Pediatrics* 1994; **40**(5): 279-84.
8. Robertson SE, Roca A, Alonso P, et al. Respiratory syncytial virus infection: denominator-based studies in Indonesia, Mozambique, Nigeria and South Africa. *Bulletin of the World Health Organization* 2004; **82**(12): 914-22.
9. Enwere G, Cheung YB, Zaman S, et al. Epidemiology and clinical features of pneumonia according to radiographic findings in Gambian children. *Tropical medicine & international health* 2007; **12**(11): 1377-85.
10. le Roux DM, Myer L, Nicol MP, Zar HJ. Incidence and severity of childhood pneumonia in the first year of life in a South African birth cohort: the Drakenstein Child Health Study. *The Lancet Global Health* 2015; **3**(2): e95-e103.
11. Hortal M, Contera M, Mogdasy C, Russi JC. Acute respiratory infections in children from a deprived urban population from Uruguay. *Revista do Instituto de Medicina Tropical de Sao Paulo* 1994; **36**(1): 51-7.
12. Cruz JR, Pareja G, de Fernandez A, Peralta F, Caceres P, Cano F. Epidemiology of Acute Respiratory Tract Infections Among Guatemalan Ambulatory Preschool Cbildren. *Review of Infectious Diseases* 1990; **12**(Supplement 8): S1029-S34.
13. Borrero I, Fajardo L, Bedoya A, Zea A, Carmona F, de Borrero MF. Acute respiratory tract infections among a birth cohort of children from Cali, Colombia, who were studied through 17 months of age. *Review of Infectious Diseases* 1990; **12**(Supplement 8): S950-S6.
14. Smith KR, McCracken JP, Weber MW, et al. Effect of reduction in household air pollution on childhood pneumonia in Guatemala (RESPIRE): a randomised controlled trial. *The Lancet* 2011; **378**(9804): 1717-26.
15. Lanata CF. Incidence and evolution of pneumonia in children at the community level. *Respiratory infections in children Benguigui Y, Lopez Antuñano FJ, Schmunis G, Yunes J, eds Washington, DC: Pan American Health Organization* 1999: 59-83.
16. Khan AJ, Hussain H, Omer SB, et al. High incidence of childhood pneumonia at high altitudes in Pakistan: a longitudinal cohort study. *Bulletin of the World Health Organization* 2009; **87**(3): 193-9.
17. Ashraf S, Huque MH, Kenah E, Agboatwalla M, Luby SP. Effect of recent diarrhoeal episodes on risk of pneumonia in children under the age of 5 years in Karachi, Pakistan. *International journal of epidemiology* 2013; **42**(1): 194-200.
18. Datta N, Kumar V, Kumar L, Singhi S. Application of case management to the control of acute respiratory infections in low-birth-weight infants: a feasibility study. *Bulletin of the World Health Organization* 1987; **65**(1): 77.
19. Pandey MR, Sharma PR, Gubhaju BB, et al. Impact of a pilot acute respiratory infection (ARI) control programme in a rural community of the hill region of Nepal. *Annals of tropical paediatrics* 1989; **9**(4): 212-20.
20. Vathanophas K, Sangchai R, Raktham S, et al. A community-based study of acute respiratory tract infection in Thai children. *Review of Infectious Diseases* 1990; **12**(Supplement 8): S957-S65.
21. Reddaiah V, Kapoor SK. Acute respiratory infections in rural underfives. *Indian journal of pediatrics* 1988; **55**(3): 424-6.
22. Pandey MR, Daulaire N, Starbuck E, Houston R, McPherson K. Reduction in total under-five mortality in western Nepal through community-based antimicrobial treatment of pneumonia. *The Lancet* 1991; **338**(8773): 993-7.
23. Zaman K, Baqui A, Sack R, Bateman O, Chowdhury H, Black R. Acute respiratory infections in children: a community-based longitudinal study in rural Bangladesh. *Journal of Tropical Pediatrics* 1997; **43**(3): 133-7.
24. Singh M, Nayar S. Magnitude of acute respiratory infections in under five children. *The Journal of communicable diseases* 1996; **28**(4): 273-8.
25. Hasan K, Jolly P, Marquis G, et al. Viral etiology of pneumonia in a cohort of newborns till 24 months of age in Rural Mirzapur, Bangladesh. *Scandinavian journal of infectious diseases* 2006; **38**(8): 690-5.
26. Broor S, Parveen S, Bharaj P, et al. A prospective three-year cohort study of the epidemiology and virology of acute respiratory infections of children in rural India. *PloS one* 2007; **2**(6): e491.
27. Brooks WA, Goswami D, Rahman M, et al. Influenza is a major contributor to childhood pneumonia in a tropical developing country. *The Pediatric infectious disease journal* 2010; **29**(3): 216-21.
28. Arifeen SE, Saha SK, Rahman S, et al. Invasive pneumococcal disease among children in rural Bangladesh: results from a population-based surveillance. *Clinical Infectious Diseases* 2009; **48**(Supplement 2): S103-S13.
29. Walke SP, Das R, Acharya AS, Pemde HK. Incidence, pattern, and severity of acute respiratory infections among infants and toddlers of a peri-urban area of Delhi: A 12-month prospective study. *International Scholarly Research Notices* 2014; **2014**.

30. Lehmann D. Tari Research Unit: Final Report for the Southern Highlands Rural Development Project: Southern Highlands Rural Development Project; 1984
31. Zhang Z, Gao L, Wang Z, Cao Y, Wu G, Zhu Z. Acute respiratory infections in Beijing children. Epidemiological studies at Dongguan Brigade. *Chin Med J (Engl)* 1986: 561-8
32. Tupasi TE, de Leon LE, Lupisan S, et al. Patterns of acute respiratory tract infection in children: a longitudinal study in a depressed community in Metro Manila. *Review of Infectious Diseases* 1990; **12**(Supplement 8): S940-S9.
33. Smith TA, Lehmann D, Coakley C, Spooner V, Alpers MP. Relationships between growth and acute lower-respiratory infections in children aged less than 5 y in a highland population of Papua New Guinea. *The American journal of clinical nutrition* 1991; **53**(4): 963-70.
34. Hu YC, Lv WY. Analysis of childhood acute respiratory infection. *Shanghai Preventive Medicine* 1996; **8**(2).
35. Sun YF, Fang XQ, He HX, Zhu QZ, Wang Q, Chen HY. Analysis of surveilance results of acute respiratory infection in children aged 0-4 years. *Maternal And Child Health Care Of China* 1992; (05): 42-5+64.
36. Zhou P, Gu MF. Surveillance of childhood acute respiratory infection in 0-4 years. *Chinese Primary Health Care* 1994; (10): 30-1.
37. Mo JZ. Surveillance of 20867 children aged 0-4 years with ARI from rural areas in South of Jiangsu. *Chinese Primary Health Care* 1998; **12**(4).
38. Xie SM, Cheng L, Hou YJ, Cao SY, Yu Q. 3097 cases of acute respiratory infection in childhood aged 0-4 years. *Chongqing Medicine* 1993; **22**(6).
39. Lou LY, Cong GQ, Sun SX, Song YH, Li GL, Yang S. Analysis of characteristics of acute respiratory infection in children younger than 15 years old in rural areas in Heilongjiang Province. *Chinese Primary Health Care* 1995; **9**(2).
40. Zhou YY, Li ZH, Chen M, Huang GH. Seasonality analysis of pneumonia incidence in children younger than 5 years old from Guangzhou. *Chinese Journal Of Child Health Care* 2000; **8**(1): 35-6.
41. Cheng P. Deceasing childhood mortality from pneumonia via applying appropriate ARI management methods. *Jian Su Journal Of Preventive Medicine* 1996; **2**.
42. Chen W, Zhao MR, Zhao YY, Ma BJ. Analysis of acute respiratory infection in children from rural areas in Henan Province. *Chinese Rural Health* 1997; **25**(1).
43. Huang WH, Chen LN, Shi YB. Situation of acute respiratory infection in children less than 5 years in Licheng District. *Strait Journal Of Preventive Medicine* 1999; (02): 21.
44. Xu GL, Zheng JY, Li LX, Wei YH, Cai ZL. Surveillance of acute respiratory infection inunder five children from Huaning County in Yunnan Province. *Chinese Primary Health Care* 2000; **14**(6): 36-7.
45. Xie YL. Research of under five childhood mortality due to pneumonia from Hubei Province. *Hubei Journal of Preventive Medicine* 2003; **14**(4): 17-9.
46. Gao JY, Feng B, Li L. Research of establishing surveillance system to monitor childhood respiratory disease to decrease the mortality from pneumonia. *Maternal And Child Health Care Of China* 2004; **19**(16): 13-4.
47. Krishnan A, Amarchand R, Gupta V, et al. Epidemiology of acute respiratory infections in children - preliminary results of a cohort in a rural north Indian community. *BMC Infectious Diseases* 2015; **15 (1) (no pagination)**(462).
